# Gleanings

**Published:** 1897-01

**Authors:** 


### GLEANINGS.

An early reduction in the price of tetanus antitoxin comes to us as news from the Pasteur Vaccine Company, a 10 c.c. bottle to cost one dollar. According to Professors Roux and Noaard, 50 c.c. should be injected as soon as possible after tetanic symptoms appear, a further injection of 20 c.c. to be given the next day, if necessary.

Streptococcic antitoxin, for use in anasarca, will shortly be placed on the list of specific curative remedies.

Powdered wood-charcoal is one of the best remedies to give to dogs that have suffered from worms and have undergone treatment for the same. It hastens a more healthful condition of the intestinal tract and tends to arrest the tendency toward their redevelopment. As its action is almost wholly mechanical, it may be given very freely.

Celluloid finely chipped and dissolved in acetone (1 : 4 by volume) is recommended as a substitute for plaster-of-Paris in bandages. Make in a close-stoppered bottle and stir well. Rub it well in after each layer of bandage. A kid glove may be used to protect the hand. It weighs but one-quarter as much as plaster-of-Paris, is very elastic, easily kept clean, and impervious to moisture.

Citrate and lactate of silver promise to be very important antiseptic agents in the treatment of wounds and for subcutaneous injection in anthrax or anasarcous conditions in equine practice. Their freely soluble nature and their non-irritant and non-poisonous properties highly commend them. Claimed to be more powerful than corrosive sublimate in the destruction of germ-life, their freedom from odor, so offensive at times with iodoform, and their ready permeability of all tissues, commend them in superiority to these last well-known agents. They may be used in powder, ointments of 1 to 50 or 100 parts of vaseline, in solution for wounds of the strength of 1 to 4000 or 5000; as a throat-spray or bath, 1 to 5000 or 10,000.

The Iceland pony mare belonging to Main's Circus recently gave birth to a colt weighing but eight and one-half pounds, measured eighteen inches from nose to tip of tail, and stood 2.3 hands high. It is said to be strong and healthy.

